# Autoregressive spatial modeling of possible cases of dengue, chikungunya, and Zika in the capital of Northeastern Brazil

**DOI:** 10.1590/0037-8682-0223-2021

**Published:** 2021-09-24

**Authors:** Silmery da Silva Brito Costa, Maria dos Remédios Freitas Carvalho Branco, Vitor Vieira Vasconcelos, Rejane Christine de Sousa Queiroz, Adriana Soraya Araujo, Ana Patrícia Barros Câmara, Angela Terumi Fushita, Maria do Socorro da Silva, Antônio Augusto Moura da Silva, Alcione Miranda dos Santos

**Affiliations:** 1 Universidade Federal do Maranhão, Programa de Pós-Graduação em Saúde Coletiva, São Luís, MA, Brasil.; 2 Universidade Federal do ABC, Programa de Pós-Graduação em Ciência e Tecnologia Ambiental, São Bernardo do Campo, SP, Brasil.; 3 Universidade Federal do Maranhão, Programa de Pós-Graduação em Saúde e Ambiente, São Luís, MA, Brasil.; 4 Secretaria Municipal de Saúde de São Luís, Vigilância Epidemiológica, São Luís, MA, Brasil.

**Keywords:** Dengue, Chikungunya, Zika, Spatial analysis, Socio-environmental factors, Economic factors

## Abstract

**INTRODUCTION:**

Dengue, chikungunya, and Zika are a growing global health problem. This study analyzed the spatial distribution of dengue, chikungunya, and Zika cases in São Luís, Maranhão, from 2015 to 2016 and investigated the association between socio-environmental and economic factors and hotspots for mosquito proliferation.

**METHODS:**

This was a socio-ecological study using data from the National Information System of Notifiable Diseases. The spatial units of analysis were census tracts. The incidence rates of the combined cases of the three diseases were calculated and smoothed using empirical local Bayes estimates. The spatial autocorrelation of the smoothed incidence rate was measured using Local Moran's I and Global Moran's I. Multiple linear regression and spatial autoregressive models were fitted using the log of the smoothed disease incidence rate as the dependent variable and socio-environmental factors, demographics, and mosquito hotspots as independent variables.

**RESULTS:**

The findings showed a significant spatial autocorrelation of the smoothed incidence rate. The model that best fit the data was the spatial lag model, revealing a positive association between disease incidence and the proportion of households with surrounding garbage accumulation.

**CONCLUSIONS:**

The distribution of dengue, chikungunya, and Zika cases showed a significant spatial pattern, in which the high-risk areas for the three diseases were explained by the variable "garbage accumulated in the surrounding environment,” demonstrating the need for an intersectoral approach for vector control and prevention that goes beyond health actions.

## INTRODUCTION

Major epidemics of dengue, chikungunya, and Zika have imposed significant social and economic costs on several countries, making these arboviruses a growing global health problem^1^. 

In the State of Maranhão, located in the northeast of Brazil, the incidences for 2016 of dengue, chikungunya, and Zika were 348.6, 195.6, and 65.5 cases per 100,000 population, respectively^2^, with the highest incidences found in the state capital, São Luís (433.6, 129.9, and 257.4 per 100,000 people, respectively).

The emergence and spread of these arboviruses depends on the presence and abundance of their main vector, *Aedes aegypti*. Despite significant efforts, the control of this vector remains a major challenge in the Americas^3^. The proliferation of the *Aedes aegypti* mosquito is influenced by complex demographic, environmental, and social factors^3^. Understanding these factors is crucial for understanding epidemics and directing control actions^3^. Urban areas with accumulated garbage and poor water supply and sanitation services are ideal breeding grounds for arbovirus vectors, and people living in these areas are more exposed to these viruses^4^. Previous studies have linked the high incidence of arboviruses to poor services in urban centers, including inadequate sanitation and water facilities and irregular garbage collection services^4^. 

The distribution of epidemic occurrence (including dengue, chikungunya, and Zika epidemics) is not homogenous in Brazil^5^. Although dengue is associated with low socioeconomic status, the evidence is conflicting^6^; some studies show a higher incidence of dengue in deprived areas^7^ ^-^ ^8^ while others report it in areas with better socioeconomic conditions^6^ ^,^ ^9^. 

Spatial modeling and geoprocessing techniques are powerful tools for understanding disease distribution. However, the spatial dynamics of the regional occurrence of dengue, chikungunya, and Zika epidemics are unclear. Studies of the spatial distribution of these diseases encompassing determining factors such as socioeconomic variables and urban infrastructure can provide a broader understanding of the dynamics of these arboviruses and produce valuable inputs to health surveillance actions^10^.

In a previous study^11^, we conducted a spatial analysis of probable cases of dengue, chikungunya, and Zika in the State of Maranhão from 2015 to 2016 using patients' municipality of residence as the unit of analysis. The present article presents a more comprehensive spatial analysis of cases in São Luís using census tracts (the smallest geographic unit for which census data are available in Brazil) and spatial and non-spatial regression models.

This study’s aim was to analyze the spatial distribution of three arboviruses (dengue, chikungunya, and Zika) across São Luís and determine the relationship between this distribution and socio-environmental and demographic factors and hotspots for mosquito proliferation.”

## METHODS

We conducted a social-ecological study of probable dengue, chikungunya, and Zika cases in São Luís, Maranhão, as recorded in Brazil's National Mandatory Reporting System (SINAN, acronym in Portuguese) from January 2015 to December 2016. The spatial units of analysis were census tracts, the smallest territorial unit in Brazil with socioeconomic and demographic data^12^.

### Study area

São Luís, the capital of the State of Maranhão, lies between the following coordinates: 02º 28' 12" and 02º 48' 09" S and 44º 10'18" and 44º 35'37" W. In 2015, the city had an estimated population of 1,073,893 inhabitants, an area of 834.785 sq. km, and a population density of 1,311.31 people/sq. km^13^. The Brazilian Institute of Geography and Statistics (IBGE, acronym in Portuguese) divides the city into 1,126 census tracts^12^.

Four tracts located in areas with mangrove swamps and dams were excluded for the purposes of this study due to the lack of population data from the 2010 Demographic Census. 

### Definition of probable cases

Probable dengue, chikungunya, and Zika cases included all cases confirmed by laboratory testing or based on clinical epidemiological criteria defined by the Ministry of Health^14^, excluding cases that tested negative or were diagnosed with other diseases^2^.

### Data collection and georeferencing

The data on probable cases were obtained from SINAN in July 2017, as described by Costa et al.^11^. The residence addresses of each case were converted into geographic coordinates using Google Maps, Bing Maps, and Wikimapia. Cases without an address or incomplete addresses were excluded. The geographic coordinates of probable cases were then converted into points in a geodatabase using the ArcGIS 10.4.1 (ESRI, Redlands, CA) geocode function. The coordinates were aggregated at the census tract level using QGIS 3.6.0, based on the map of São Luís obtained from IBGE's website and the 2010 census tract grid. 

### Calculation of incidence rate

The outcome of interest was the census tract dengue, chikungunya, and Zika incidence rate, calculated as the ratio between the combined number of probable cases of the three diseases in the census tract from 2015 to 2016 (numerator) and the tract population (denominator), multiplied by 100,000 inhabitants. The combined number of cases was used because, as described by Rodrigues et al.^6^, the diseases are transmitted by the same vector and have clinical similarities that can lead to diagnostic, and therefore, notification errors. For the tract populations, we used data from the 2010 census obtained from the IBGE website^12^. 

### Study covariates

The following socio-environmental and demographic data were obtained from the 2010 Demographic Census, conducted by the IBGE: population density; average household income; illiteracy rate; literacy rate; subnormal clusters (areas with poor living conditions); proportion of households with no public water supply; proportion of households without waste collection services; proportion of households with an accumulation of garbage in the surrounding environment; and proportion of households exposed to open sewers in the surrounding environment^12^. 

Hotspots were defined as key properties or areas, such as scrap metal and recycling yards, tire repair shops, building material warehouses, gas stations, and cemeteries, that facilitate the proliferation of *Aedes aegypti* because of the large number of objects that accumulate rainwater and provide potential mosquito breeding grounds. According to the national dengue control and prevention guidelines, hotspots for mosquito proliferation should be identified, registered, and inspected in two-week cycles to perform larval assessment and focal/residual treatment^15^.

The data on hotspots for mosquito proliferation sites were provided by the city council's epidemiological surveillance department on a spreadsheet containing the description of each property. Of the 1,164 hotspots identified by the city council during the study period, 698 were geocoded and 466 were discarded (313 because of incomplete addresses and 153 because they were duplicated). The geocoded sites were then aggregated by census tract.

### Spatial and statistical analysis

Local empirical Bayes estimates were used to reduce random variations in incidence rates. Smoothed local rates are more stable because they consider both the population of the tract and that of neighboring tracts^16^. 

Spatial autocorrelation of the incidence rate and the pattern of spatial distribution and census tract clustering intensity were measured using Global Moran's I and Local Moran's I (both adjusted for population density^17^ ^,^ ^18^), respectively. These algorithms combine the Moran indices with their respective Bayesian smoothing standardization and are better recommended for spatial analysis of risk rates^19^. Different orders of contiguity were tested for the neighboring matrix by selecting the order that best demonstrated the spatial autocorrelation structure. A significance level of 5% was used for both indices. Global Moran's I adjusted for population densities was validated using a pseudo-significance test with 999 permutations. The results from the Local Moran's I test adjusted for population densities were expressed on a local index space association (LISA) cluster map.

For the non-spatial and spatial regression models, we used the Naperian log (Ln) of the Bayesian smoothed rate as the dependent variable to make the distribution of the dataset more consistent with a normal distribution. For the multiple linear regression model (ordinary least squares regression), the independent variables were selected using stepwise regression, adopting a cutoff value of 0.05.

Two spatial autoregressive models (spatial lag and spatial error) were applied to incorporate spatial effects. These models seek to capture the spatial correlation structure in a single global parameter that is analogous to the Global Moran Index and add it to the classic regression model^18^. The independent variables included in the spatial models were the same as those used in the multiple linear regression model. 

The first spatial model (1) we used was the *spatial lag model*, which considers spatial dependence by adding a new term to the regression model in the form of a spatial relation to the dependent variable.

Y=Xβ+ρ WY+ε (1)

Here, *X* is the matrix of the explanatory variables, *ρ* is the spatial autoregressive coefficient, *W* é is a spatial proximity matrix*, WY* expresses spatial dependence on *Y,* and *ε* is the error term^17^. 

The second model (2) was the *spatial error model*, in which the effects of spatial autocorrelation are associated with the error term *ε*. This model considers spatial effects as perturbations that should be removed.

Y=Xβ+ε,ε=λWε+ξ (2)

Here, 𝑊𝜀 is the error component with spatial effects, *λ* is the autoregressive coefficient, and 𝜉 is the uncorrelated component with constant variance^17^.

Model performance was assessed using the likelihood function. The model with the best fit was determined using the Akaike information criterion (AIC) and Bayesian information criterion (BIC)^18^. The residuals of the models were analyzed using Global Moran I and Local Moran I to test whether spatial autocorrelation was eliminated after applying the models. 

The open-source Geographic Information System QGIS 3.6.0 was used to cluster the cases by census tract and GeoDa 1.14 was used to calculate the incidence rate, local empirical Bayes estimates, and Global Moran I and Local Moran I, and to run the spatial autoregressive models. Multiple linear regression was performed using Stata® 14.0. (College Station, TX, USA).

### Ethical aspects

This study was approved by the Research Ethics Committee of the University Hospital at the Federal University of Maranhão (code number: 2.228.632).

## RESULTS

From 2015 to 2016, a total of 5,124, 1,419, and 2,855 cases of dengue, chikungunya, and Zika, respectively were reported in São Luís. We excluded 448 (8.65%), 53 (3.73%), and 635 (22.42%) cases of dengue, chikungunya, and Zika, respectively, because of missing information regarding the patient’s residential address. Finally, 4,681, 1,366, and 2,220 probable cases of dengue, chikungunya, and Zika, respectively were included, summarizing a total of 8,267 cases.

**FIGURE 1:**
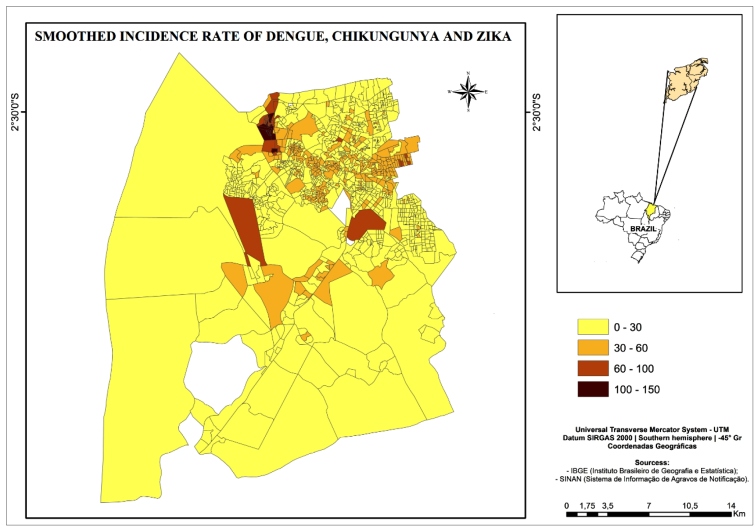
Spatial distribution of smoothed incidence rate of dengue, chikungunya and Zika. São Luís, MA, 2015-2016.

Analysis of the smoothed incidence rates showed that rates were higher in the west, northeast, and center of the city and surrounding areas (Figure 1).

**FIGURE 2:**
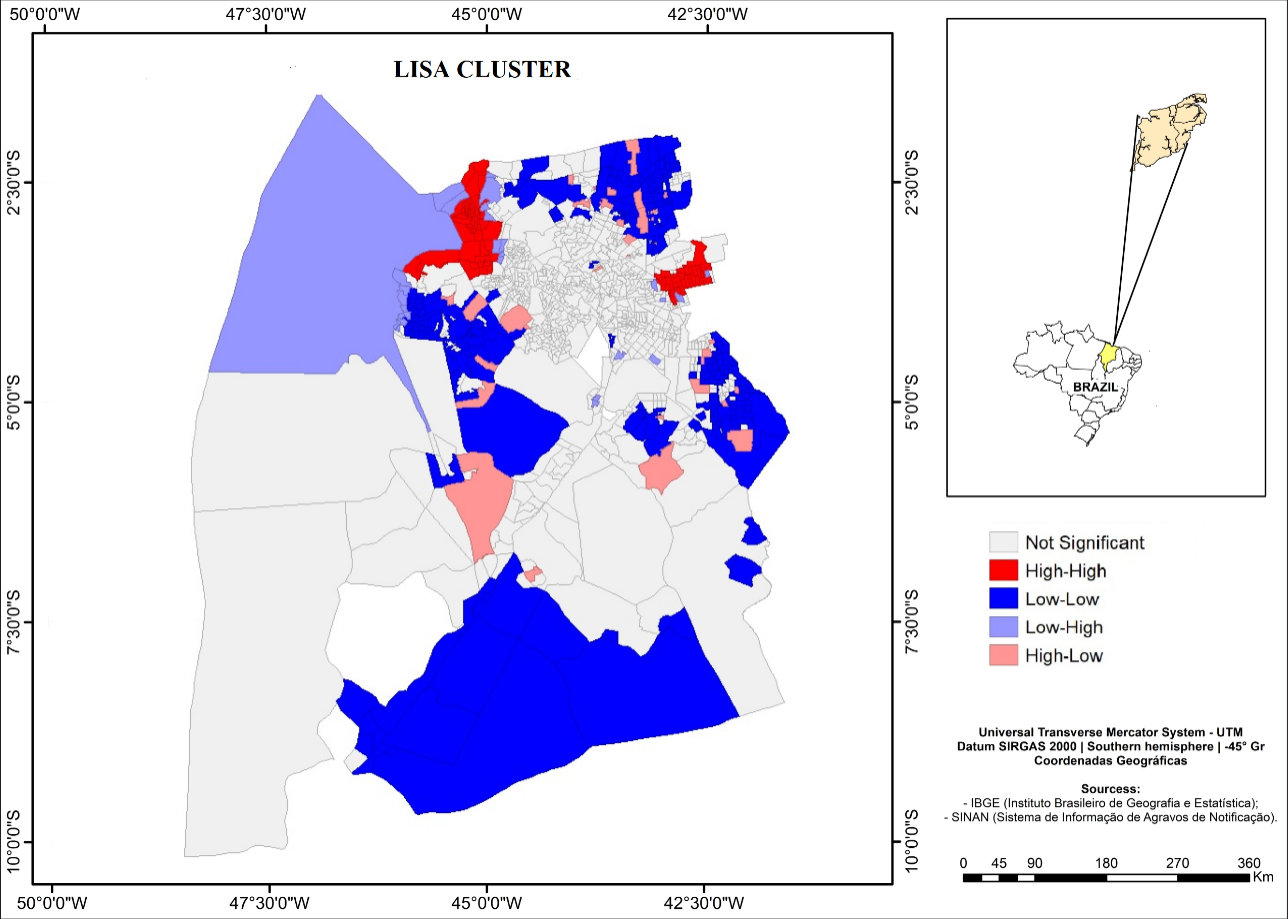
Lisa Cluster Map of Smoothed incidence rate of dengue, chikungunya and Zika. São Luís, MA, 2015-2016.

The second-order queen contiguity neighbor matrix was selected because it best demonstrated the spatial autocorrelation structure in Moran's I tests. The Global Moran's I test showed a statistically significant positive spatial autocorrelation for the raw and smoothed incidence rates (I=0.32; p=0.001 and I=0.54; p=0.001). Local Moran's I (Figure 2) showed hotspots with blocks of census tract clusters with high smoothed incidence rates and neighbors with high incidence in the western region, where the São Francisco neighborhood is located, and in the northeast region, where the Cohab and Cohatrac neighborhoods are located. These neighborhoods are located in health districts that show historical dengue incidence rates^20^. Low-incidence clusters were found in the northeast, west, southeast, and east regions.

In the multiple linear regression model, the population density, subnormal clusters, hotspots for mosquito proliferation, literacy rate, and proportion of households without waste collection services were eliminated by stepwise analysis. In the final multiple linear regression model, the dependent variable showed a positive relationship with the proportion of households that had accumulated garbage in the surrounding environment (p<0.0001) and a negative relationship with illiteracy, income, proportion of households exposed to open sewers in the surrounding environment, and proportion of households with no public water supply (Table 1).

**TABLE 1:** Final multiple linear regression model for the log of the smoothed incidence rate in São Luís, Maranhão, Brazil, 2015-2016.

Independent variables	Coefficient	Standard error	T*	P-value
Illiteracy	-0.0523058	0.0119235	-4.38679	0.00001
Income	-6.07528e-005	1.84072e-005	-3.3005	0.00100
Accumulation of garbage	0.00463741	0.00177366	2.6146	0.00905
Open sewers	-0.047704	0.0161893	-2.94664	0.00328
No public water supply	-0.00504505	0.000764192	-6.60181	0.00000

*T-test.

The spatial lag model showed the best fit when compared to the multiple linear regression and spatial error models, obtaining the highest values for the likelihood function and explanatory power (R^2^=0.508) and lowest values for AIC and BIC (Table 2). The only variable that maintained a positive statistically significant relationship (p=0.03) after running this model was the proportion of households with an accumulation of garbage in the surrounding environment (correlation coefficient=0.26; standard error=0.13).

**TABLE 2:** Comparison between the spatial and regression models of data from São Luís, Maranhão, Brazil, 2015-2016.

	Multiple Linear Regression	Spatial Lag Model	Spatial Error Model
R^2^	0.088100	0.508086	0.507973
Likelihood function	-1323.34	-1021.64	-1025.38
Akaike Information Criterion	2658.68	2059.28	2064.77
Bayesian Information Criterion	2688.78	2099.46	2099.92
Moran’s I - Residual*	0,37	- 0,000	-0,001

*Global Moran of the residue of three utilized models.

**FIGURE 3:**
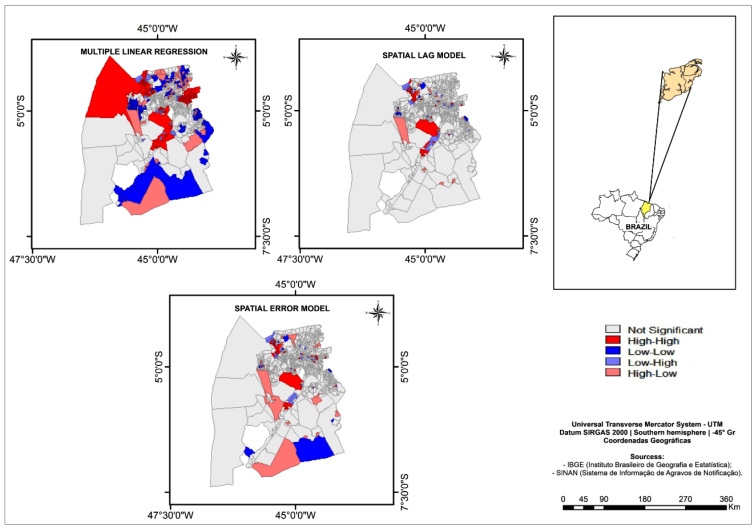
Lisa Cluster Map of the residuals from the Multiple Linear Regression model, Spatial Lag Model and Spatial Error Model. São Luís, MA, 2015-2016.

The Global Moran's I test showed that the spatial lag and spatial error models eliminated the spatial autocorrelation of the residuals (I=-0.000, p=0.48, and I=-0.001, p=0.40, respectively). The LISA cluster map (Figure 3) shows randomness in the spatial distribution of the residuals, especially in comparison to the residuals from the multiple linear regression model.

## DISCUSSION

The distribution of dengue, chikungunya, and Zika cases in São Luís showed a significant spatial autocorrelation, with a predominance of high incidence clusters in the west and northeast of the municipality. From an epidemiological perspective, this pattern may be due to the existence of proliferation hotspots and mosquito movement, focal areas of virus transmission, and the spatial autocorrelation of underlying explanatory factors. 

Chikungunya and Zika were emerging diseases in Brazil during the study period, with a particularly high number of cases in the northeast region of Brazil. The number of reported cases of dengue also increased significantly across the country during the same period. The most prevalent dengue serotype during this period was DENV-1^2^. Herd immunity may limit the transmission of dengue serotypes in some tracts. Although it is unknown which serotype circulated in 2016, DENV-1 and DENV-2 are known to have circulated in São Luís during 2015. However, reports show that all four dengue serotypes circulated in the city prior to 2015. The fact that chikungunya and Zika were emerging diseases during the study period means that the population was particularly vulnerable to infection.

The negative linear relationship between illiteracy and disease occurrence shown by the multiple linear regression model may be linked to the underreporting of diseases, which may be more pronounced among populations with a lower level of education. Other studies using multiple regression analysis in the states of Rio de Janeiro and Minas Gerais^21^ ^,^ ^22^ reported a significant negative association between dengue incidence and illiteracy rates, corroborating this hypothesis. Strong similarities between the spatial pattern of education and other variables such as income and basic sanitation (proportion of households exposed to open sewers in the surrounding environment and those with no public water supply) may influence the results of statistical models that do not incorporate the spatial structure of data. For example, in a classic case of multicollinearity, higher-income areas are likely to have better sanitation and low illiteracy rates, making it difficult to determine the separate and joint influence of these factors on the dependent variable in these areas. 

The global spatial effects models performed better than the multiple linear regression model, showing that considering spatial dependence can help considerably in understanding disease occurrence patterns. This is shown by the spatial autocorrelation of the dependent variable, confirmed by the Global Moran's I and Local Moran's I and by the increase in the explanatory power of the variables shown by the R^2^ value and other model quality indicators.

The values of the residuals from the Moran's I tests do not show spatial dependence, demonstrating the efficiency of the spatial models. The spatial models showed a better fit to the data than the non-spatial models, as shown in other studies^8^ ^,^ ^23^. Spatial models better explain the relationship between factors and the incidence of diseases such as dengue because they capture inequalities across geographical spaces^10^. 

The best performing model was the spatial lag model, with only the proportion of households with an accumulation of garbage in the surrounding environment remaining in the final model*.* Similarly, a study conducted in the State of Minas Gerais reported an association between waste management and the incidence of dengue, chikungunya, and Zika. This association was particularly pronounced for dengue, suggesting that adequate waste management protects against dengue^24^. Contrastingly, a study in Rio de Janeiro^6^ found no relationship between the risk of infection and basic sanitation factors.

Previous studies conducted in São Luís^25^ and in Niterói^26^ report that garbage was accumulated around houses, vacant lots, and irregularly occupied areas in these cities despite regular waste collection services. Other studies in Thailand^27^ and Indonesia^28^ showed that open-air waste disposal significantly influenced the risk of developing dengue. A study in São Luís^29^ reported that all deaths due to dengue between 2002 and 2013 were among patients who lived in census tracts with poor waste collection services.

Small containers found in household waste are shown to fill up quickly with rainwater, becoming potential breeding grounds considering the short time it takes for larvae to develop into adult mosquitoes^30^. The high incidence of arboviruses^31^ may, therefore, be related to the presence of garbage containers close to households.

A survey of basic sanitation conducted by the IBGE in 2019 showed that the disease most reported by local councils was dengue and that disease occurrence was associated with, among other factors, garbage accumulation in households and the street^30^ ^,^ ^32^. Although the population is often aware of vector control measures, this knowledge rarely results in the adoption of preventive practices^33^. A study in São Luís reported a gap between knowledge and practice in the form of behavioral changes among a large part of the population, illustrated by an increase in the use of non-recyclable materials, improper disposal of household waste, throwing of garbage in vacant lots, and the existence of large quantities of breeding sites for *Aedes aegypti* in households^25^. The effective participation of the community in the elimination of breeding grounds and health surveillance actions is, therefore, vital to the success of epidemic control and prevention programs^33^.

One of the risk areas for dengue, chikungunya, and Zika identified in this study was the neighborhood Cohab. Silva et al.^34^ reported the existence of informal dumping grounds, improper commercial and household waste disposal, and irregular waste collection services in this neighborhood. Informal dumping grounds lack adequate environmental and health protection measures^35^. This situation highlights the importance of environmental education in promoting adequate waste disposal and avoiding environmental pollution^34^.

It is important to understand how health relates with the environment to promote effective prevention of arbovirus epidemics^4^. Public health managers should promote an intersectoral approach to tackle the multiple determinants of health^33^. Vector control should extend beyond health actions to include policies that encompass social mobilization, environmental education, and improvements in housing conditions and sanitation^36^.

No significant relationship was found between hotspots for mosquito proliferation and the dependent variable. This may be the result of effective vector control at these sites, meaning that they had a limited influence on the incidence of these arboviruses. In the second half of 2016, the Municipal dengue Control Program launched the campaign “*selo legal*”, a label awarded to establishments that meet mosquito control and prevention criteria designed to promote the reduction of vector infestation rates at mosquito proliferation hotspots^37^. 

In Cubatão, Guarujá, Praia Grande, Santos, and São Vicente in the State of São Paulo, a reduction in control activities at hotspots for mosquito proliferation led to an increase in the abundance of *Aedes aegypti*, reinforcing the importance of continuity in control interventions for vector elimination^38^.

Figure 3 showing the clustering of the residuals from the spatial lag model may be used to identify census tracts in which high levels of occurrence of dengue, chikungunya, and Zika cases are not explained by neighbor structure or the independent variable (accumulated garbage). These areas may contain proliferation hotspots that have yet to be identified or lack effective control and prevention actions, and, therefore, warrant special attention. This may be the case of the hotspot with high incidence rates noted in the São Francisco neighborhood, which appears as a high-high cluster in Local Moran's I map (Figure 2), but still shows a cluster of high positive residuals in the autoregressive models. Contrastingly, the hotspot of high incidence in the northeast region (Cohab and Cohatrac neighborhoods, Figure 2) is well explained by the autoregressive models, showing no significant cluster of residuals; therefore, the high incidence rates there may be due to the high proportion of households with an accumulation of garbage in the surrounding environment, which was a significant explanatory variable in these models.

Study limitations include possible underreporting of cases, possible differential reporting across census tracts, inherent limitations to ecological studies, geocoding inaccuracies, spatial resolution in the census tract, homogenizing possible intra-tract heterogeneity in disease incidence and socioeconomic characteristics, and the fact that the presence of asymptomatic and subclinical cases precludes an accurate evaluation of real incidence. Finally, the high percentage of losses due to incomplete addresses may have biased the association with socioeconomic factors, as addresses are more likely to have not been geolocalized in poor areas.

The study’s strengths are as follows: only few studies have conducted a spatial analysis of the three arboviruses (dengue, chikungunya, and Zika) in conjunction^6^ ^,^ ^11^ ^,^ ^24^ ^,^ ^39^ ^,^ ^40^, priority areas for prevention and control interventions were identified using spatial analysis, underlying socio-environmental factors were investigated using regression and spatial models, and the unit of analysis used enabled a more detailed analysis of clustering. 

In the final spatial model, disease incidence was explained by the accumulation of garbage in the surrounding environment. This finding demonstrates that the control of these arboviruses extends beyond health management, requiring integrated actions across other sectors and services, such as urban cleaning, infrastructure, and waste management, including awareness-raising and behavioral changes to ensure proper waste disposal and reduce the proliferation of *Aedes aegypti*.
